# The Role of Pathology in the Diagnosis of Swine Respiratory Disease

**DOI:** 10.3390/vetsci8110256

**Published:** 2021-10-29

**Authors:** Giuseppe Sarli, Giulia D’Annunzio, Francesca Gobbo, Cinzia Benazzi, Fabio Ostanello

**Affiliations:** Department of Veterinary Medical Sciences, University of Bologna, 40064 Bologna, BO, Italy; giulia.dannunzio2@unibo.it (G.D.); francesca.gobbo3@unibo.it (F.G.); cinzia.benazzi@unibo.it (C.B.); fabio.ostanello@unibo.it (F.O.)

**Keywords:** PRDC, respiratory disease, diagnosis, swine, histopathology

## Abstract

The definition “porcine respiratory disease complex” (PRDC) is used to indicate the current approach for presenting respiratory pathology in modern pig farming. PRDC includes pneumonias with variable pictures, mixed with both aerogenous and hematogenous forms with variable etiology, often multimicrobial, and influenced by environmental and management factors. The notion that many etiological agents of swine respiratory pathology are ubiquitous in the airways is commonly understood; however, their isolation or identification is not always associable with the current pathology. In this complex context, lung lesions registered at slaughterhouse or during necropsy, and supplemented by histological investigations, must be considered as powerful tools for assigning a prominent role to etiologic agents. In recent years, the goal of colocalizing causative agents with the lesions they produce has been frequently applied, and valid examples in routine diagnostics are those that indicate pulmonary involvement during porcine reproductive and respiratory syndrome virus (PRRSV) and porcine circovirus type 2 (PCV2) infections.

## 1. Introduction

Respiratory disease is one of the main causes of production losses in the global swine industry, and porcine respiratory disease complex (PRDC) is a primary source [1]. PRDC is a multifactorial disease caused mainly by the interaction of bacterial and viral pathogens and, less frequently, by parasites also influenced by environmental and management stressors and pig-specific factors. In PRDC, several viral and bacterial agents can be detected in various combinations, resulting in polymicrobial infection [2,3]. Therefore, the agents involved can act both as primary and as secondary pathogens (Table 1). The onset of respiratory disease on swine farms is thought to be generally related to a primary viral insult that promotes a secondary bacterial infection [1].

Numerous primary viral pathogens such as porcine reproductive and respiratory syndrome virus (PRRSV) [4,5], porcine circovirus type 2 (PCV2) [6,7], swine influenza virus (SIV) [8], pseudorabies virus (PRV) [9,10], and porcine respiratory coronavirus (PRCV) [11,12] are endemic in pig farms (Table 1). However, the viral pathogens associated with respiratory diseases in pigs vary significantly between farms, production sites, regions, and countries, making generalization difficult [13]. Although they can cause severe disease on their own, more often, uncomplicated infections with these agents are mild and transient [9].

Even though many potential bacterial pathogens colonize the nasal cavity or tonsils of pigs, normal respiratory defense mechanisms prevent damage or spread to the lungs. Hence, based on their ability to damage the upper airway epithelium, injure the lung parenchyma, and promote secondary bacterial colonization, the primary viral pathogens are able to influence the development and outcome of PRDC [13]. Some bacterial agents such as *Mycoplasma hyopneumoniae* and *Pasteurella multocida* [14], *Streptococcus suis* [10,15], and *Actinobacillus pleuropneumoniae* [16] (Table 1) may act as both primary and secondary invaders depending on the situation. It is when these primary infections become complicated with secondary bacteria that more serious and chronic respiratory diseases occur and the most economic loss is incurred.

Determining whether viruses or bacteria are responsible for a swine respiratory problem can be difficult. In fact, other considerations limit diagnosis on a clinical basis in the following ways: (1) The disease due to the virus is overshadowed by the effect of concurrent or secondary bacterial infections; and (2) the clinical signs are shared among different viruses affecting the respiratory system, and differences may be subtle, inconsistently present, or less prominent than the effect of secondary bacterial infections [17].

The complexity of the above-indicated interactions between pathogens makes it difficult to study PRDC, the prevention and control of the disease [1], and the identification of the etiologic agent responsible for a respiratory episode [18]. Pig respiratory diseases mostly include rhinitis, pneumonia, and pleuritis, which may occur as a result of association or not [9].

The aim of this review is to highlight the role and the limits of gross and microscopic pathology, to provide an etiologic indication in the diagnosis of the primary swine respiratory diseases, with a particular focus on the epidemiological situation in European countries. 

## 2. Rhinitis

**Figure 1:**
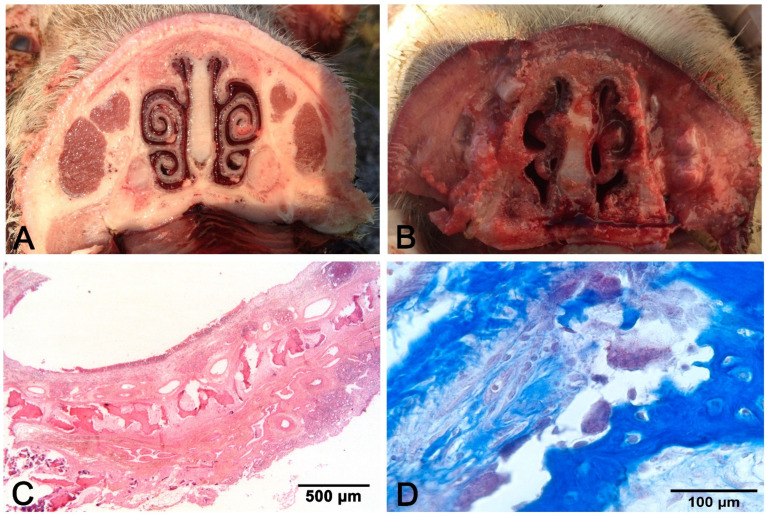
Progressive atrophic rhinitis (PAR): (**A**) control non-affected; (**B**) bilateral symmetric complete atrophy of turbinates; (**C**) discontinuous mural bone in the turbinate; (**D**) hyperplasia of osteoclasts facing bone trabeculae. (**C**) hematoxylin–eosin (H–E) stain. (**D**): Masson’s trichrome stain.

Rhinitis is very common in piglets; sneezing and oculonasal catarrhal to purulent discharge are the main symptoms [9]. Two main types of rhinitis are known in swine: nonprogressive atrophic rhinitis (NPAR) and progressive atrophic rhinitis (PAR). Both can affect the tropism of turbinates, but the effect is considered mild and transient in NPAR, while long-lasting and more severe forms allow the twisting (in the case of asymmetric turbinates atrophy) or shortening (in the case of bilateral turbinate lesions) of the snout in PAR [19]. Transverse sections of the snout at the level of the first or the second upper premolar allow the appreciation of the turbinate atrophy in PAR (Figure 1), which usually starts in the ventral and then moves to the dorsal turbinate. PAR histology evaluates osteoclast hyperplasia and osteoclast-mediated bone resorption, followed by the replacement with fibrous tissue [19] (Figure 1). 

The etiology of NPAR encompasses viruses (Cytomegalovirus, SIV, PRV), bacteria (*Bordetella bronchiseptica*), and environmental contaminants (high concentrations of NH_3_, dusts) [9], while the etiology of PAR includes a synergistic interaction between *B. bronchiseptica* infection followed by the action of a heat-labile toxin produced by strains A and D of *Pasteurella multocida* [20]. Because gross lesions of NPAR and PAR are qualitatively similar, although those of NPAR are generally mild, the identification of *B. bronchiseptica*, and of toxigenic strains of *P. multocida*, is imperative for PAR diagnosis [19]. Both PAR and NPAR predispose an animal to pneumonia. 

Another form of swine rhinitis is inclusion body rhinitis due to cytomegalovirus—for which histology is useful in terms of detection—as well as inflammation, basophilic nuclear inclusion bodies associated with karyomegaly and cytomegaly in the epithelial cells of the nasal mucosal glands [19].

## 3. Pattern of Pneumonia

Pathogens responsible for respiratory diseases reach their targets by aerogenous or hematogenous routes that, in the lungs, develop different patterns and distributions of the lesions. 

### 3.1. Macroscopic Pattern of Aerogenous Lung Involvement

**Figure 2:**
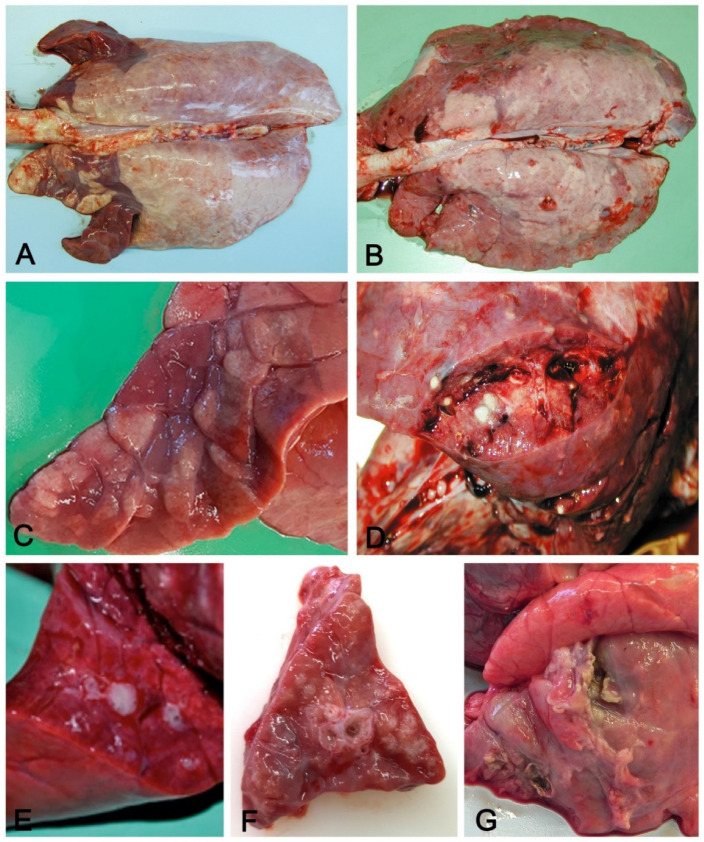
Aerogenous lung involvement. Cranioventral distribution (**A**) of coalescing, slightly depressed dark-red lobules (**C**) with consolidation and mucus in airways (**E**) in an acute, moderate, aerogenous non-complicated pneumonia. These features are indicative of enzootic pneumonia. Cranioventral distribution of consolidated dark-red lung parenchyma (**B**) with multifocal yellowish foci of suppuration (**D**) with pleural ulceration and fibrosis (**G**). These features are indicative of complicated enzootic pneumonia or of bronchopneumonia. In (**F**), cuffing of small airways, usually present in enzootic pneumonia, but also found in complicated pneumonias.

Aerogenous lung involvement includes both upper and lower airways and alveolar inflammation, while the extrapulmonary airway-only pathology is rarely reported in swine. Szeredi et al. [21] describe acute tracheal edema and hemorrhage accompanied by fibrinonecrotic tracheitis without detectable pathogens or associated pulmonary pathology, and they speculate on a pathogenetic mechanism similar to bovine Honker syndrome. Aerogenous lung involvement induces a cranioventral pattern of which examples are swine enzootic pneumonia (EP) and bronchopneumonia. Respiratory viruses (e.g., PRV, SIV) and *M. hyopneumoniae*, often associated with secondary bacterial agents, are the causes. Gross features of aerogenous pneumonias, summarized in Figure 2, include:

1. Variation in consistency (from consolidation in acute stages to fibrosis when chronicization occurs);
2. Color variation: from dark red in consolidated areas, to a whitish color in chronic lesions. The chromatic variation may also be accentuated by inflammatory edema (acute phase) of the perilobular connective tissue and by its fibrous thickening (chronic phase);
3. Presence in the airways, from scarce and dense catarrhal exudate (in EP caused by *M. hyopneumoniae*) to collection of pus in complicated EP and in bronchopneumonia;
4. Whitish mural thickening (cuffing pneumonia) of small airways present in EP.

Distribution of the lesions can be lobular, involving single or coalescing consolidated lobules or lobar when an entire lobe is involved with acute (an example is the fibrino-necrotizing pneumonia caused by *A. pleuropneumoniae*) or chronic lesions (progressive involvement of the whole lobe in complicated EP and in bronchopneumonia, with the presence of both acute and recent lobular lesions intermingled with chronic fibrotic or purulent changes). In EP, typical fissures are found, i.e., deepening of the parenchyma due to collapsed alveoli or to interlobular fibrous thickening [19]. 

**Figure 3:**
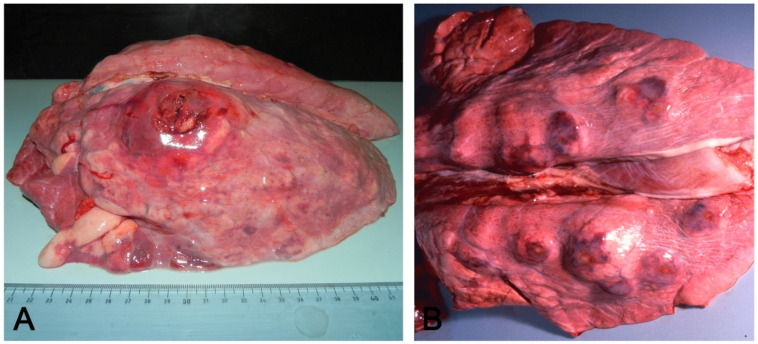
Acute (**A**) and chronic (**B**) pleuropneumonia by *Actinobacillus pleuropneumoniae*: in (**A**), an acute, locally extensive, and protruding nodule coexists with associated pleuritis localized in the craniodorsal side of the left lung lobe, referable to *A. pleuropneumoniae* and cranioventral pneumonia. In (**B**), multifocal nodules of the chronic form.

There are some exceptions, however. *A. pleuropneumoniae* reaches the lung through the airways, but bronchitis is not a typical feature, except in complicated cases. The lesions are localized in the dorsal portions of the caudal lung lobes [9], and acute fibrinosuppurative, hemorrhagic, and necrotizing lobar pneumonia, with consolidated lung and fibrinous pleuritis or chronic features (a large unilateral area or multiple foci of fibrosis, necrosis or its suppuration), are frequent (Figure 3).

Another exception is pneumonia caused by SIV: the pathogenesis is aerogenous, but the lesion is a tan consolidation of cranial lobes [19] associated with multifocal lobular consolidation of the caudal lobes [22].

### 3.2. Macroscopic Pattern of Hematogenous Lung Involvement

**Figure 4:**
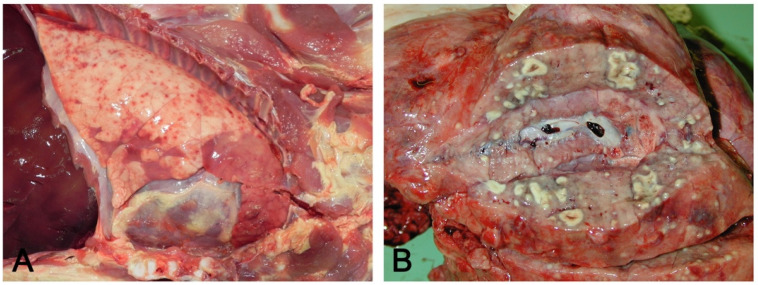
Hematogenous lung spread: in (**A**), together with a cranioventral pneumonia, the right basal lobe shows multiple acute and recent red foci of hyperemia/hemorrhage referable to bacteremia often of intestinal origin. In (**B**), multiple foci of suppuration surrounded by normal lung parenchyma, due to lung arrest of microthrombi originating from septic phlebitis outside the lungs.

**Figure 5:**
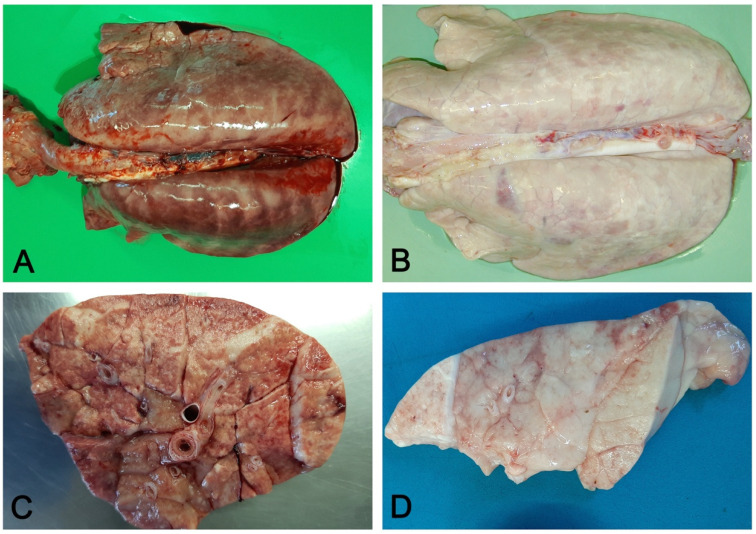
Interstitial pneumonia: non-collapsed lungs, with rib impressions (**A**), in acute (**A**) and chronic (**B**) interstitial pneumonia. Acute changes include diffuse hyperemia and edema of perilobular connective tissue (**C**). In chronic stages, the lung is slightly increased in consistency and colored whitish due to fibrosis (**D**).

If etiological agents enter the lung through the blood, lesions can involve the whole organ; they will primarily be located in the dorsal areas of the caudal lobes where vascularization is more prominent. Hematogenous lung involvement may appear as:

- Embolic pneumonia (arrest in the lung of septic thromboemboli originated from inflammation located elsewhere). Its prominent macroscopic feature is the presence, if recent, of randomly scattered foci of hyperemia/hemorrhage or, if chronic, of abscesses (Figure 4).
- Interstitial pneumonia (arrival of pneumotropic or endotheliotropic agents from primary sites of replication through the blood circulation as in PRRSV and PCV2 infections and septicemia from Gram-negative bacteria). The macroscopic appearance is an interstitial pneumonia characterized by lungs that fail to collapse, with rib impressions, patchy, lobular or diffuse distributions of color variation (ranging from red in the acute stages to a pale whitish color in the chronic forms), changes in consistency (firm texture), interstitial edema (in acute stage), and missing airway involvement (Figure 5).

Pulmonary hematogenous pathology also has examples in *Metastrongylus* spp. and *Ascaris suum* pulmonary invasion, in which interstitial pneumonia is due to alveolar–septal damage by larvae and, only for *Metastrongylus*, by adults located in small bronchi in the dorsal areas of the lung, where they cause catarrhal–purulent bronchitis to which bacterial complications contribute. These latter lesions are complex, as they are sustained by both interstitial and granulomatous pneumonia directed against parasites. They are associated with emphysema and atelectasis whether the presence of exudate and parasites in the small airways causes obstruction or occlusion, respectively.

In both aerogenous and hematogenous forms of lung involvement, other lesions can include enlarged tan mediastinal lymph nodes. In pseudorabies, pigs may occasionally exhibit inflamed turbinates or trachea, or necrosis in the tonsils or trachea [17]. PRCV does not usually induce distinctive gross lesions [17]. In the hematogenous spread of systemic diseases (PCV2 and PRRSV), other sites, mainly lymphoid organs, are targeted by specific lesions [19].

Attempts to correlate the viruses and/or bacteria identified with the lung gross lesions classified as catarrhal bronchopneumonia, purulent bronchopneumonia, interstitial pneumonia, broncho-interstitial pneumonia, pleuropneumonia, and pleuritis have yielded significant results, but a single agent has been related to more than one type of lesion [23]. This can be influenced by the simultaneous presence of different viruses and/or bacteria in the etiopathogenesis of the disease [23].

Although the macroscopic pattern of airborne or hematogenous lung involvement is different, secondary bacterial complications cause the gross appearance to be shared between the two. Histology, in these cases, can provide further discriminating details. Histology and agent identification are also necessary in cases of macroscopic evidence of EP lesions, because this macroscopic feature often represents a mild and initial pneumonia in swine to an agent different from *M. hyopneumoniae* [19].

### 3.3. Molecular Diagnostic Tools

PRDC is characterized by a significant overlap in clinical presentation and pathological lesions, and the identification of the causative agents requires the integration of clinical information with gross and microscopic findings and laboratory analysis [9].

A wide range of direct or indirect diagnostic tests may be used when PRDC is suspected. Enzyme-linked immunosorbent assays (ELISA), indirect fluorescent antibody tests (IFA), and immunoperoxidase monolayer assays (IPMA) are frequently used to detect the presence of specific antibodies against some infectious agents [24,25].

For the detection of the majority of bacterial respiratory diseases, bacterial culture is the most common, but not necessarily the most rapid, diagnostic method. However, bacterial culture allows one to evaluate the antimicrobial susceptibility of the isolated bacteria [25]. Virus isolation (VI) can be used to grow and identify viruses from clinical samples, but it requires freshly submitted samples and it may require two or more weeks to obtain results. For these reasons, the direct diagnosis of viral infections is currently performed with molecular methods such as polymerase chain reaction (PCR), quantitative PCR (qPCR), or multiplex PCR [25,26,27,28]. Due to its polymicrobial nature, rapid identification of the causative agent in PRDC is complicated by the absence of a single diagnostic test routinely used for the detection of the major PRDC-associated pathogens [29]. Therefore, over time, multiplex PCR or one-run real-time PCR detection systems have been developed and tested for pathogens associated with respiratory diseases in pigs [18,29].

First- or second-generation sequencing (NGS) complements detection by PCR, further confirming the identification of the pathogen of interest and also characterizing it at the strain level [25]. 

Although PCR is a sensitive method, it does not discriminate infection from disease, and the detection of an infectious agent does not necessarily make it the cause of the disease [30]. 

The rapid diagnosis by molecular methods allows for the detection of a pathogen during infection, while serological tests are an appropriate approach for the monitoring of infections in a herd [31]; other promising approaches are oral fluids (OF) tests and other non-conventional samples, such as processing fluids (PF) [28]. 

Table 2 reports the samples to collect in cases of pathogens with only respiratory or systemic tropism.

### 3.4. Histopathology and Lesion–Etiology Colocalization

**Figure 6:**
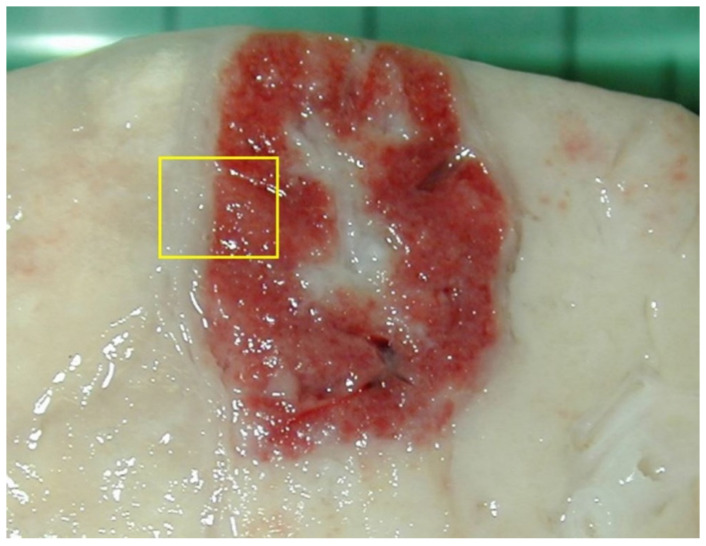
For histological examination, the lesions should never be collected in the center, since they often contain a complicated or necrotic lesion. A sample at the margin with the surrounding normal parenchyma (yellow box) allows one to identify the pathological process at its onset (in the peripheral part of the lesion) and its evolution (progressing in observation towards the center). If the lesions are small (1–2 cm), they can be sampled entirely; if they are larger, it is appropriate to take them from the margin, with some surrounding normal tissue.

Histopathological tools can be an important diagnostic support for identifying the pathogens involved in diseases. PRRSV, *Glaeserella (Haemophilus) parasuis*, and *Streptococcus* spp. in weaned piglets and PRRSV, *P. multocida*, and *Streptococcus* in fattening pigs were frequently identified in lungs without gross lesions [23]. This strengthens the importance of associating the identification of a pathogen with the lesion produced. Microscopic examination of the tissues, in some cases, allows for the observation of the characteristic morphological changes induced by a specific pathogen, and microscopy can be a quick assay for diagnosis [32]. Samples should be obtained from the most recent and acute lesions, where the probability of colocalizing the responsible pathogen and the lesions it produces is greater. Consequentially, sampling of internal areas rich in necrosis or with chronic features should be avoided (Figure 6).

Histologic lesions are sometimes suggestive of the responsible organism.

**Figure 7:**
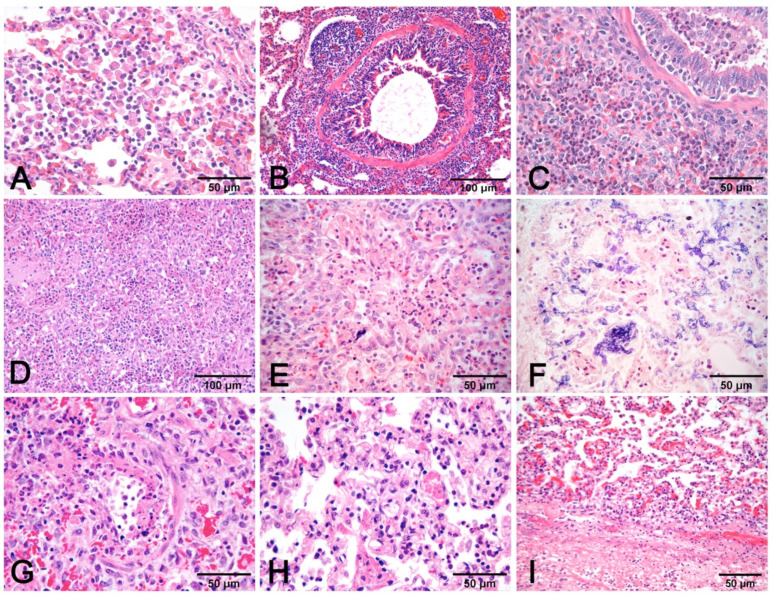
Pneumonia with alveolar macrophages (**A**) and lymphocytic peribronchial cuffing (**B**); when associated with gross changes in cranioventral non-complicated pneumonia, these histological findings are indicative of EP. Increase in airway and alveolar neutrophils (**C**) indicates complication. Necrosis of lung parenchyma (**D**) and of alveolar cell exudate (**E**), fibrin alveolar collection (**F**) associated with vessel fibrinoid necrosis (**G**), thrombosis (**H**), and fibrinous pneumonia (**I**) are a histologic indication of porcine pleuropneumonia, for which etiologic indication is imperative, as similar gross and histologic changes are also due to bacteria different from *Actinobacillus pleuropneumoniae*. (**A**–**E**,**G**–**I**): H–E stain. (**F**): PTAH stain.

**Figure 8:**
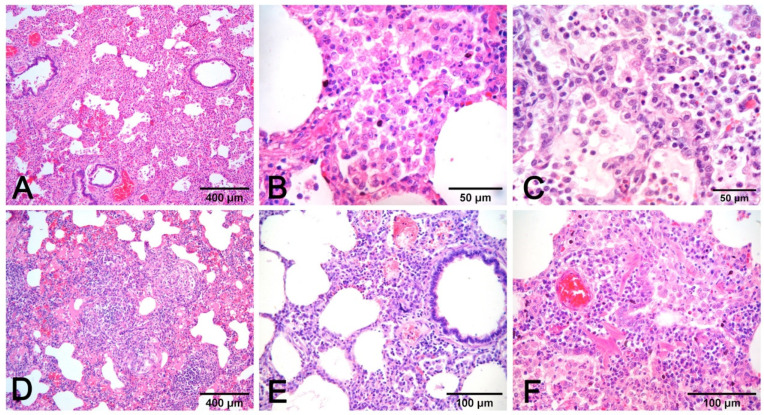
Interstitial pneumonia in PRRSV (**A**–**C**) and PCV2 (**D**–**F**) infections share similarities that need etiologic validation. A granulomatous pattern of inflammation (**D**) is suggestive of PCV2 pneumonia, whereas it is lacking in PRRSV pneumonia. This latter infection is characterized by macrophage alveolar exudation and necrosis (**B**) and type II pneumocyte hyperplasia (**C**) in alveolar walls. In PCV2 pneumonia, both alveolar septa and peribronchial space are thickened (**E**), and bronchiolar necrosis (**F**) can be recognized. H–E stain.

- Alveolar exudates, mainly represented by edema and macrophages associated with mucus in airways surrounded by lymphocytes to cause peribronchial cuffing, are strongly suggestive of non-complicated EP [19]. The presence of alveolar neutrophils and fibrin is indicative of bacterial complication (Figure 7A–C).
- Necrosis of airway epithelium with cellular debris and leucocytes filling the lumen and broncho-interstitial pneumonia are the hallmarks of SIV infection [19]. Necrosis should also be considered in PRCV infections, where it is limited to small and terminal bronchioles [17], and in PCV2 infections [19] (Figure 8F).
- Fibrinous or necrotizing pneumonia with alveolar necrotic (mainly neutrophils) leucocytes and fibrin collection, associated with vascular thrombosis and fibrinoid mural necrosis, often with concurrent fibrino-hemorrhagic pleuritis, is highly indicative of peracute/acute *A. pleuropneumoniae* infection (Figure 7D–I). In these cases, the isolation/identification of the etiologic agent is imperative, as the same histologic pattern can be attributed to *Actinobacillus suis*, septicemic salmonellosis, and pleuritic strains of *P. multocida* [9,19].
- Interstitial pneumonia, characterized by variable thickening of bronchiolar and alveolar walls, is a hallmark of viral infections:
  ○ It is generally mild in PRCV and from mild to severe in PCV2 and PRRSV infections;
  ○ Interstitial pneumonia in PRRSV infection is characterized by type 2 pneumocytes hyperplasia and intra-alveolar necrotic macrophages. It is often associated with prominent rhinitis [17] (Figure 8A–C);
- Pneumonia in PCV2 infection is characterized by peribronchial space and alveolar septa thickened by lymphocytes and macrophages, with a pattern of interstitial to granulomatous pneumonia (Figure 8D,E). Among porcine circovirus diseases (PCVD), lung lesions are reported in both systemic (PCVD-SD; PMWS) and in lung diseases (PCVD-LD); however, when lung lesions are detected, those cases are more often PCVD-SD than PCVD-LD [33]. Other features are bronchiolar necrosis (Figure 8F) progressing to bronchiolitis obliterans and lymphocyte depletion or granulomatous lymphadenitis in regional lymph nodes or other lymphoid tissues [19].
- PRV does not usually induce recognizable microscopic lesions in the lung of older pigs; however, in a few pigs, usually younger, focal necrosis of the parenchyma may be present. Vasculitis, focal cerebral gliosis, and nonsuppurative meningitis in the brain are more consistent lesions [17]. PRV may induce prominent rhinitis.

Proliferative and necrotizing pneumonia (PNP) is a histologic pattern characterized by the alveolar collection of macrophages and necrotic debris, associated with the hyperplasia of type II pneumocytes and thickening of alveolar septa by inflammatory cells [19]. This pattern is shared by several pathogens (Table 1) such as PRRSV and PCV2 (often coexisting) infections, but also PRV and SIV [34,35,36,37]. 

Histopathology makes diagnosis easier in cases of infection with pathogens difficult to cultivate in vitro [38,39], since it can be supported by non-specific histochemical stains, such as acid-fast staining for *Mycobacteria* spp. and *Nocardia* spp., or Gram staining for other bacteria [32,40]. Histopathology has high specificity techniques available such as immunohistochemistry (IHC) or in situ hybridization (ISH), which, respectively, label the antigen or the nucleic acid of an infectious agent [32]. 

**Figure 9:**
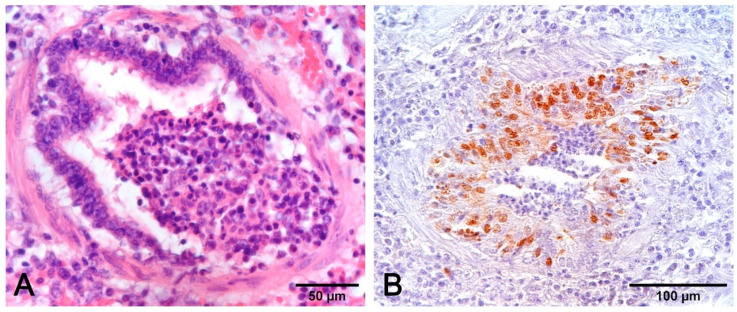
Airways epithelium necrosis (**A**) is a hallmark of SIV infection, but the lesion is shared with other pathogens and the acquisition of etiologic indication is imperative. The opportunity to have positive results by IHC (**B**) is contingent on time, because the lungs can rapidly clear the virus. In this case, sampling on animals with very recent and acute symptoms, in association with other investigations (PCR, serology), provides useful data ((**A**): H–E stain; (**B**): IHC with monoclonal anti-SIV antibody clone 1331. Meridian Life Science, Inc.).

The sensitivity of histological tools for assessing a pathogen (IHC and ISH) is lower than other tests (e.g., PCR), and the amount of pathogens in tissue is not always identifiable in histologic sections. Usually, mainly acute, pathogen-rich lesions are more prone to providing positive histologic results. Two other main limits should be taken into account in the histologic assessment of pathogens: (1) pathogen persistence; for example, lung tissues are easily cleared of SIV and samples can be negative from 72 h post infection [19]; and (2) the availability and specificity of primary reagents (antibodies, probes) for the investigated pathogens and their ability to work with formalin-fixed tissues (Figure 9). 

Immunohistochemical detection of an antigen in tissue samples also denotes the distribution of pathogens in the organ/body and contributes to pathogenesis studies in both field and experimental trials [41,42,43]. IHC and ISH procedures to detect PRRSV, PCV2, and SIV [8,44,45,46] were used to investigate their role in finishing pigs with respiratory disease [14,26,35,47], as well as in experimental reproduction of the disease and in coinfection models [48,49,50,51].

**Figure 10:**
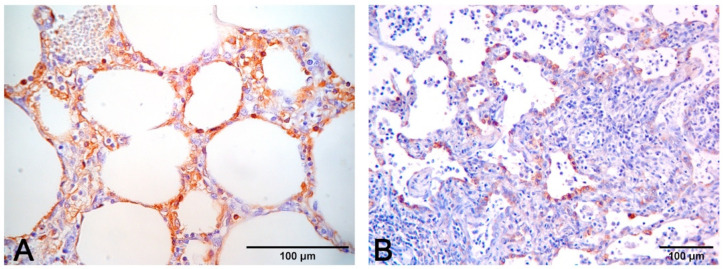
(**A**) Immunohistochemical stain to PCV2 (monoclonal antibody anti-PCV2, clone F217) with positive macrophages in alveolar septa. (**B**) Immunohistochemical stain to PRRSV (monoclonal antibody anti-PRRSV, clone SDOW17-A) with positive hyperplastic type II pneumocytes and rare interstitial machrophages.

The presence of bronchointerstitial pneumonia and PCV2 antigen in infiltrating macrophages is indicative that PCV2 plays a role in the etiology of pneumonia [52,53]. In the lung, the PCV2 antigen or genome can be detected mainly in the cytoplasm of macrophages and dendritic cells, but also in monocytes, bronchial epithelial cells, and endothelial cells [33,54,55]. The combination of IHC and histology also allows to visualize the PRRSV antigen in the cytoplasm of alveolar macrophages, type II pneumocytes, and in bronchial epithelial cells [35,56,57] (Figure 10). 

Histopathology is also a support in the study and characterization of the pathogenicity of new viral variants and their variation in cellular adaptation and tropism [58,59,60].

Histopathological tools can also be used to detect bacterial nucleic acids or antigens, for example, to diagnose respiratory mycoplasmosis or *A. pleuropneumoniae* infections [61,62,63]. In the lung, *M. hyopneumoniae* is localized on the surface of bronchial and bronchiolar epithelial cells [64], while *A. pleuropneumoniae* binds to respiratory epithelial cells of the terminal bronchioles and to pneumocytes [10].

Histopathology has also found application in vaccine efficacy evaluation studies, with the aim of detecting viral/bacterial tissue residue after natural or experimental infections following a vaccine challenge (e.g., PRRSV, PCV2, *M. hyopneumoniae*) [64,65,66,67,68,69,70,71].

## 4. Pleuritis

**Figure 11:**
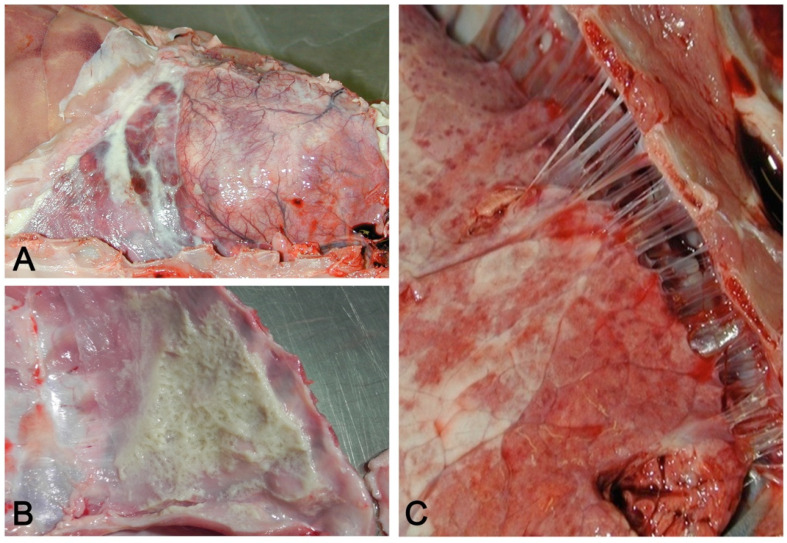
(**A**) Acute serofibrinous pleuritis and pericarditis: fibrinous exudate forms loosely adherent strands of material on the pleural surface. (**B**) Layers of fibrin covering the thoracic wall in acute serofibrinous pleuritis. (**C**) Chronic fibrous pleuritis with strongly adherent fibrous strands between the lung and thoracic wall.

Pleuritis may be associated with pulmonary disorders (a classic example is in fibrinous pneumonia by *A. pleuropneumoniae*). In pigs, pleuritis often may not be concomitant with pneumonia, but instead, with other serosites (pericarditis, peritonitis) and arthritis in systemic pathology, supported etiologically by *G. parasuis*, *Streptococcus suis*, *Mycoplasma hyorinis*, and *Actinobacillus* spp. [9]. Pleural involvement can follow an underlying lung pathology (abscess or bronchopneumonia) or hematogenous dissemination of bacteria, in which frank pneumonia is lacking. The most frequent types of pleuritis in swine are acute sero-fibrinous (Figure 11A,B), followed by chronic fibrous pleuritis with multiple adherences (Figure 11C), after organisation of the fibrin. Pleuritis is a common finding in swine at slaughterhouses; chronic fibrous pleuritis is associated with serologic positivity to *A. pleuropneumoniae* and is concluded to be the etiology in most cases, particularly those with a dorsocaudal pleural surface involvement [72].

## 5. Conclusions

Macroscopic patterns of lung involvement and the histologic characterization of lesions are the strongest signs that pathology provides in addressing a diagnosis. Both indications, however, need to be integrated with other direct or indirect methods to assess etiology, with clinical history and epidemiological data. Pathology contributes to direct or indirect diagnostic methods when antibodies or probes are available to objectivate the presence of viruses or bacteria in histologic sections. This latter tool allows for the colocalization of an agent inside the lesions and is the main goal already applied in some diseases (PCV2 and PRRSV infections); however, this approach must be used for other diseases in the near future. Among the limits of the diagnostic direction that pathology can provide are the complications that reduce the reliability of macroscopic and microscopic patterns. This bias can be reduced by a proper sampling on animals with recent and acute lesions or by integrating with the pathology other investigations in cases of chronic and complicated lesions.

## Figures and Tables

**Table 1:** Primary and secondary pathogens in porcine respiratory disease complex (PRDC) [9].

Primary Pathogens	Secondary Pathogens
PRRSV * SIV * PRCV PCV2 * Mycoplasma hyopneumoniae *Actinobacillus pleuropneumoniae*	*Mycoplasma* spp. Streptococcus spp. Staphylococcus spp. *Escherichia coli* Klebsiella spp. *Trueperella pyogenes* *Bordetella bronchiseptica* *Glaesserella parasuis* *Pasteurella multocida*

* Association with swine proliferative and necrotizing pneumonia (PNP).

**Table 2:** PRDC: specimen collection (modified from [9,17]).

Suspected Etiology/Disease ^a^		Tissue/Sample	Fresh/Fixed (10% Buffered Formalin)
*Systemic tropism* e.g., PCV2 PRRSV PRV *M. hyorinis*		Lung	From affected areas with different gross appearance (cranial, middle lobes and the cranial portion of the caudal lobe with visible airways)
		Lymph nodes	Mandibular, sternal, tracheobronchial, mesenteric and superficial inguinal
		Tonsil	One or both sides
		Spleen	Representative portion or in case of absence of lesions elsewhere
		Heart (PCV2; *M. hyorinis*)	Left and right ventricles and septum
		Brain (PRV)	Representative portion or in case of absence of lesions elsewhere
*Respiratory* *tropism* e.g., NPAR and PAR *M. hyopneumoniae* *A. pleuropneumoniae*	Upper respiratory tract	Nasal turbinate	1 cm thickness
	Low respiratory tract	Lung	From affected areas with different gross appearance (cranial, middle lobes and the cranial portion of the caudal lobe with visible airways)

^a^ Pathogens with only respiratory tropism reach the target by the aerogenous route and are only manifested in the airways/lung of the pathology. In such cases, airways/lung sampling (for etiological or histological investigations) represents an indispensable and sufficient target. Pulmonary inflammatory diseases with hematogenous pathogenesis have, in the lung, the extension of disease localized in other organs that are often more suitable for sampling to obtain the necessary diagnostic indications. In such cases, attention should be focused on the lung, but also on the lymphoid organs (PCV2, PRRSV) and extrapulmonary septic processes at different locations, which, in addition to the results obtained from lung sampling, have greater value in providing etiological indications.

## Data Availability

The study did not report any data.
